# Evaluation of Trigeminocardiac Reflex in Patients Undergoing Elevation of Zygomatic Fractures

**DOI:** 10.7759/cureus.22281

**Published:** 2022-02-16

**Authors:** Priyanka A Mhamunkar, Vinayakrishna Kolari, Joyce Sequeira

**Affiliations:** 1 Oral and Maxillofacial Surgery, Yenepoya Dental College, Mangalore, IND

**Keywords:** oculo-cardiac reflex, zygoma fracture, zygomatic complex fracture, aschner dagini reflex, trigeminocardiac reflex

## Abstract

Aim

Trigeminocardiac reflex (TCR) manifests as typical hemodynamic perturbations including a sudden lowering of heart rate, mean arterial blood pressure (MABP), cardiac arrhythmias, and asystole. In literature, TCR has been seen during ocular surgeries, Lefort fractures, and craniofacial surgeries. However, the prevalence of the TCR has not been studied in zygomatic complex fractures . The aim of this study was to assess the prevalence of TCR in patients undergoing elevation with/without fixation of zygomatic complex fractures and isolated zygomatic arch fractures under local anesthesia and general anesthesia and to evaluate the prevalence of TCR in different age groups.

Materials and methods

The study comprised 26 participants diagnosed with zygomatic fractures indicated for surgical intervention. The aim of the study was to find the prevalence of TCR in patients undergoing surgical intervention (elevation of zygomatic complex fractures with/without fixation) under local anesthesia and general anesthesia. The heart rate and blood pressure were measured preoperatively, intraoperatively and postoperatively.

Results

Variation in heart rate was seen in patients undergoing surgery under local anesthesia and general anesthesia. However, a decrease in the heart rate i.e., bradycardia was noted intra-operatively in 75% of the patients operated under local anesthesia. The prevalence of TCR was noted intra-operatively in 23% of cases operated under general anesthesia. No significant changes were seen in the blood pressure of the patients.

Conclusion

In our study, we found out that the prevalence of TCR was more in the patients operated under local anesthesia i.e., 75% of patients. Out of the patients operated under general anesthesia i.e., 23% of patients showed TCR. No significant variations in blood pressure were observed in patients operated under local anesthesia or general anesthesia. The prevalence of TCR was found more often in the age group of 31-45 years in our study.

## Introduction

Trigeminocardiac reflex (TCR) which was traditionally known as the oculocardiac reflex is a rare physiologic phenomenon that can be seen during a surgical procedure in the maxillofacial region. TCR was first observed by Kratschmer in 1870 [1]. Later, in 1908 a peripheral variant of TCR was observed by Aschner and Dagini and hence was then popularly known as Aschner-Dagini Reflex. It occurs as a result of pressure effects in the distribution of the trigeminal nerve, specifically the ophthalmic division of this branch. Any pressure changes in the distribution of the trigeminal nerve cause the afferent neurons to carry the impulse to the trigeminal nucleus. Here, the short internuncial fibers carry the efferent impulse to the vagus nerve, thus stimulating it causing bradycardia and gastric hypomobility [2]. Hence, while manipulating the fractures of the mid-face there elicits a reflex resulting in bradycardia and asystole. The aim of this study was to assess the prevalence of TCR in patients undergoing elevation with/without fixation of Zygomatic complex fractures/arch fractures, under local anesthesia and general anesthesia. We also aimed to evaluate the prevalence of TCR in different age groups. As there are less data available in the literature about this reflex, we decided to carry out this study for a better understanding of this physiological phenomenon.

## Materials and methods

A descriptive study was planned and carried out in the Department of Oral and Maxillofacial Surgery, Yenepoya Dental College and hospital, Yenepoya University, Mangalore between December 2019 - July 2021 on 26 patients diagnosed with zygomatic complex fracture. The Yenepoya Ethics Committee 2 approved the study (YEC2/486). The aim of this study was to assess the heart rate of the patient to see the prevalence of TCR and to assess the changes in the blood pressure in the patients undergoing elevation with/without fixation of zygomatic complex fractures/arch fractures under local anesthesia and general anesthesia. A total of 26 participants were included in the study who were diagnosed and operated on for zygomatic complex fracture. 

The inclusion criteria were patients with zygomatic complex fractures with/without other facial fractures and isolated zygomatic arch fractures, patients within the age group of >15years and <60 years, and patients who were willing to participate in the study. The exclusion criteria were patients taking medications for an underlying cardiac ailment, patients taking oral anticoagulants, and pregnant patients. Consent was obtained from all the willing participants in the language deciphered by them. The assessment was done using blood pressure and heart rate recording machine (Contec Multipara patient monitor CMS 8000). Blood pressure and heart rate were recorded preoperatively (five minutes before placing the incision), intraoperatively (during the elevation of the fracture), and postoperatively (five minutes after suturing). Patients were divided into age groups 15-30 years, 31-45 years, 46-60 years to evaluate the prevalence of TCR in different age groups post-data collection. All the records were maintained in a Microsoft Excel sheet. Heart rate less than 60 beats per minute qualified as bradycardia in our study according to the criteria given by Meuwly et al. [2].

## Results

**Figure 1:**
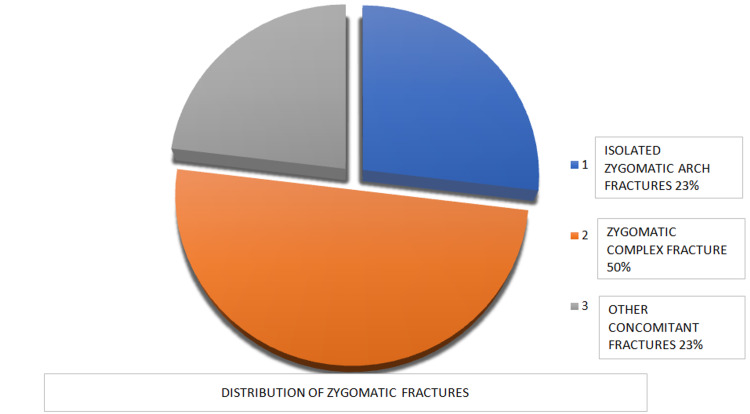
Pie chart describing the distribution of the zygomatic fractures in the study sample

In our study, a total sample of 26 patients was evaluated for TCR. Out of 26 samples collected, eight were operated under local anesthesia i.e., 30.7%, and 18 were operated under general anesthesia i.e., 69% of the study population (Tables 1-2). Out of the 26 samples, 24 were males i.e., 92% were males and two were females i.e., 7.6%. The average age of the study population was 33.7 years, with an average age of males being 32 years and females being 48 years. Out of the 26 samples of zygomatic fracture in our study, seven were isolated zygomatic arch i.e., 26%, 13 were zygomatic complex fractures i.e., 50%, and six were zygomatic bone fractures with other fractures of the face i.e., 23% (Figure 1).

**Table 1:** Table showing the demographic details of the patients operated under general anesthesia, their heart rate (HR) and blood pressure (BP) recorded preoperatively, intraoperatively, postoperatively

No.	AGE	SEX	TYPE OF FRACTURE	TREATMENT DONE	TYPE OF ANAESTHSIA	PRE-OP BP	PRE-OP HR	INTRA-OP BP	INTRA-OP HR	POST-OP BP	POST-OP HR
1	20	Male	Left ZMC fracture	Open reduction	GA	140/90	96	138/90	87	134/86	87
2	43	Male	Left ZMC fracture	Open reduction	GA	126/70	73	127/61	54	118/67	57
3	34	Male	Bilateral lefort, left ZMC, left supra orbital	Open reduction	GA	109/69	84	110/77	66	110/73	82
4	35	Male	Left ZMC fracture	Closed Reduction	GA	116/76	78	112/78	72	110/73	66
5	45	Male	Right ZMC fracture	Open reduction	GA	159/105	71	145/80	85	149/88	94
6	31	Male	Left lefort 3, left ZMC, left orbital floor, nasal and right subcondylar fracture	Open reduction	GA	131/78	74	128/72	74	132/78	72
7	19	Male	Right incomplete lefort an left ZMC an lefort	Open reduction	GA	121/71	88	133/84	73	144/86	75
8	37	Male	Left ZMC fracture	Open reduction	GA	134/74	76	102/63	63	109/73	69
9	23	Male	Left ZMC, left angle, right parasymphysis	Open reduction	GA	128/95	104	101/88	92	117/86	95
10	54	Male	RIGHT ZMC, NO	Open reduction	GA	126/98	77	118/98	78	128/97	78
11	24	Male	Right ZMC fracture	Open reduction	GA	116/77	72	116/78	72	118/75	74
12	33	Male	Right ZMC fracture	Open reduction	GA	101/75	78	102/72	58	113/70	85
13	23	Male	Left ZMC fracture	Open reduction	GA	98/64	72	95/64	72	103/61	85
14	24	Male	Left ZMC fracture	Closed Reduction	GA	109/69	72	100/47	83	103/51	66
15	33	Male	Left ZMC fracture	Closed Reduction	GA	107/72	64	124/84	71	104/78	58
16	44	Male	Right ZMC fracture	Closed Reduction	GA	96/63	79	121/80	79	116/78	62
17	25	Male	Right ZMC, right subcondylar and dentoalveolar	Open Reduction	GA	130/68	68	110/72	60	106/70	72
18	51	Female	Incomplete ZMC fracture	Closed reduction	GA	135/77	102	126/74	102	162/74	103

**Table 2:** Table showing demographic details of the patients operated under local anesthesia (LA), their heart rate (HR) and blood pressure (BP) recorded preoperatively, intraoperatively, postoperatively

No.	AGE	SEX	TYPE OF FRACTURE	TREATMENT DONE	TYPE OF ANAESTHSIA	PRE-OP BP	PRE-OP HR	INTRA-OP BP	INTRA-OP HR	POST-OP BP	POST-OP HR
1	35	Male	Right Zygomatic arch fracture	Closed reduction	LA	138/72	72	138/84	66	130/68	72
2	50	Male	Right Zygomatic arch fracture	Closed reduction	LA	110/78	68	110/70	52	106/70	62
3	37	Male	Left Zygomatic arch fracture	Closed reduction	LA	130/68	69	160/90	60	148/50	70
4	56	Male	Right Zygomatic arch fracture	Closed reduction	LA	112/72	65	110/72	53	110/73	50
5	45	Female	Right Zygomatic arch fracture	Closed reduction	LA	138/72	76	120/74	54	130/68	76
6	45	Male	Left Zygomatic arch fracture	Closed reduction	LA	110/78	89	120/74	60	120/78	80
7	36	Male	Right ZMC and arch fracture	Closed reduction	LA	132/92	92	128/80	54	132/98	96
8	30	Male	Right zygomatic arch fracture	Closed reduction	LA	110/70	86	130/90	70	120/90	72

**Figure 2:**
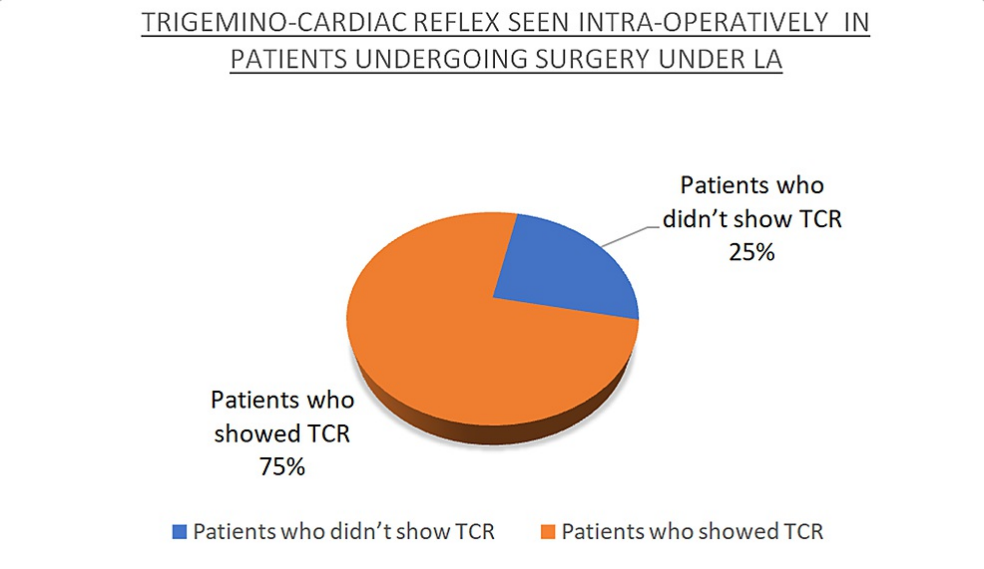
Pie chart describing prevalence of trigeminocardiac reflex (TCR) in patients undergoing surgery under local anesthesia

**Figure 3:**
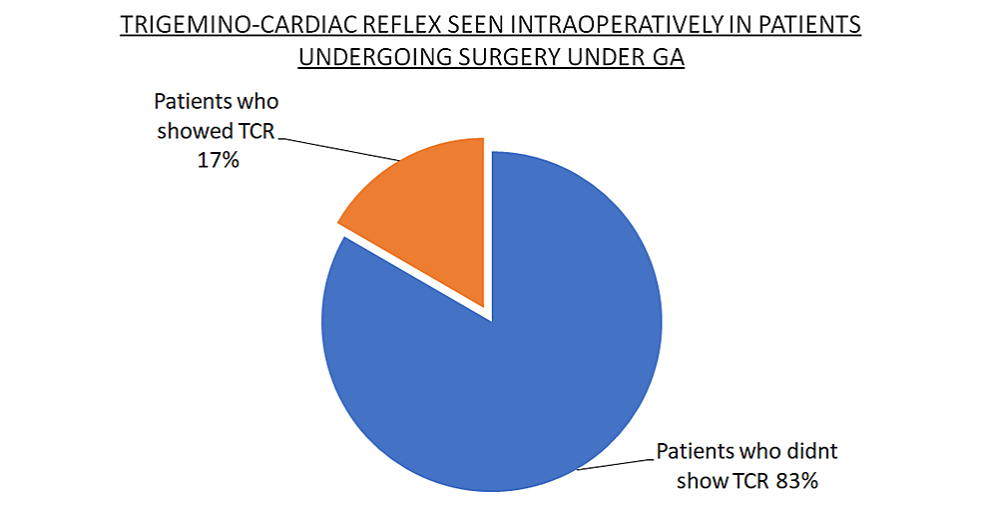
Pie chart describing prevalence of trigeminocardiac reflex (TCR) in patients undergoing surgery under general anesthesia

The average heart rate recorded preoperatively in the patients who underwent surgery under local anesthesia was 77 beats/minute, intraoperatively 50 beats/minute, and postoperatively 72 beats/minute. The average blood pressure noted in patients who underwent surgery under local anesthesia was preoperatively is 138/85 mm Hg and intraoperatively 110/75 mm Hg and postoperatively 140/82 mm Hg. The average heart rate noted preoperatively in the patients who underwent surgery under general anesthesia was 76 beats/minute, intraoperatively was 74 beats/minute and postoperatively was 76 beats/minute. The average blood pressure noted in the patients who underwent surgery under general anesthesia preoperatively was 135/81 mm Hg and intraoperatively 120/78 mm Hg and postoperatively 134/82 mm Hg. Out of the eight patients operated under local anesthesia, six patients i.e., 75% patients showed bradycardia indicative of TCR intraoperatively (Figure 2). Whereas out of the 18 patients with zygomatic complex fracture operated under general anesthesia, only three patients i.e., 17% showed TCR (Figure 3).

The lowest pulse recorded was 50 beats/minute, post-operatively in the patient operated under local anesthesia. The highest pulse recorded was 103 beats/minute in the patient operated under general anesthesia post-operatively. One patient in the age group of 15-30 years who was being operated under general anesthesia developed TCR intraoperatively. Two patients who were operated under general anesthesia and four patients who were operated under local anesthesia in the age group of 31-45 years developed TCR intraoperatively. Two patients in the age group of 46-60 years who were operated under local anesthesia developed TCR intraoperatively. Thus in our study, the prevalence of TCR was found to be more in the age group of 31-45 years. Significant changes were not observed in the blood pressure of the patients undergoing surgery for zygomatic fractures under local anesthesia or general anesthesia. All the patients exhibiting TCR were managed conservatively by promptly stopping the treatment momentarily until the heart rate was stabilized. No additional medical management with anti-cholinergic drugs was required. All the patients exhibited normal heart rate postoperatively.

## Discussion

TCR, a sudden decrease in pulse rate i.e., bradycardia and mean arterial blood pressure (MABP) but also apnoea and gastric hypermotility during stimulation of any branches of the trigeminal nerve. The pressure induced by the neural reflexes on the trigeminal nerve causes vagal stimulation leading to bradycardia [1,2]. In 1987, Bainton and Lizi [3], and Loewinger et al. [4], suggested that TCR can be elicited by stimulating afferent paths other than the ciliary nerves (branches of the ophthalmic division of the trigeminal nerve) which are classically associated with the oculocardiac reflex . It varies according to the type of stimulus and location. In the literature, 90% of TCR occurs most frequently in ophthalmic surgery, followed by 8%-18% in skull base surgery and the least incidence in craniofacial surgery, 1%-2%. During the elevation of zygomatic arch fracture; two cases of bradycardia were reported by Loewinger et al. in 1987 [4] and Shearer et al. in 1987 [5]. It was also noticed in orthognathic surgery, TMJ surgery, and reconstruction surgery [6].

In our study, the heart rate was recorded at three different intervals intraoperatively. According to Meuwly et al., it was considered bradycardia when the heart rate was <60 beats per minute or 20% of the baseline heart rate. In our study, heart rate < 60 beats/min was considered as bradycardia [2]. As zygomaticomaxillary complex fractures are anatomically in the vicinity of the ophthalmic branch of the trigeminal nerve, we thought of independently assessing the prevalence of TCR. Recording the heart rate and blood pressure of the patient at three different intervals during the course of the surgery to give us an idea regarding the sudden onset of bradycardia during the elevation of the fracture segment which might lead to TCR. In our study, we observed bradycardia intraoperatively, during the elevation of the fracture segment. This could be suggestive of the fact that during the elevation of the fracture, pressure changes occur that cause the initiation of this reflex. TCR was observed more in the patients operated under local anesthesia as compared to the patients operated under general anesthesia. TCR is believed to be non-fatal in the intraoperative scenario but can cause arrhythmias and that should be of clinical concern to us. Usually, intraoperative bradycardia can be corrected with anticholinergic drugs like atropine 1 mg IV which is given over 3-5 minutes to normalize the heart rate but, in our study, the treatment was stopped momentarily which resulted in the spontaneous return of heart rate to normalcy without any medical aid [7-9]. Blood pressure changes were not significant in our study. The age group of 31-45 was thought to be more susceptible to develop TCR due to high vagal tone and increase in the level of anxiety of the patients in this particular age group. It was also suggested by Guedes et al. that TCR can be observed 24 hours after the procedure, but as the heart rate and blood pressure were not recorded beyond the intraoperative hours, we could not assess if TCR can occur as a delayed reflex [10]. Though the literature shows many studies showing the TCR, the actual pathophysiology is still unclear, and hence more studies need to be done to clearly understand this phenomenon [11-14]. TCR is further classified as territorial reflex and extraterritorial reflex. Territorial reflex which includes the TCR that occurs due to the stimulation of the opthalmic, maxillary, and mandibular division of the trigeminal nerve whereas extraterritorial reflex includes the reflex that occurs due to trigeminal nerves anatomical considerations [15].

The drawback of our study was the smaller sample size; drawing a conclusion on a physiological phenomenon is not definitive with this smaller sample size. A study with a larger sample size would be more conclusive. Also, anatomically similar fractures were not included in the study, which could cause bias. As heart rate and blood pressure were not recorded for a longer duration, the prevalence of delayed TCR was not assessed in our study.

## Conclusions

In our study, we found that the prevalence of TCR was more in the patients operated under local anesthesia i.e., 75% patients. Out of the patients operated under general anesthesia i.e., 23% of patients showed TCR. No significant variations in blood pressure were observed in patients operated under local anesthesia or general anesthesia. The prevalence of TCR was found to be more in the age group of 31-45 years in our study. This study will help in better understanding of TCR phenomenon, its pathophysiology, and the clinical outcome of this reflex. By understanding the predisposing factors, the anatomical considerations in the maxillofacial region that cause this reflex, one can promptly identify the reflex and timely intervention can minimize the morbidity/mortality associated with it.
